# Laccase-Catalyzed Surface Modification of Thermo-Mechanical Pulp (TMP) for the Production of Wood Fiber Insulation Boards Using Industrial Process Water

**DOI:** 10.1371/journal.pone.0128623

**Published:** 2015-06-05

**Authors:** Mark Schubert, Pascal Ruedin, Chiara Civardi, Michael Richter, André Hach, Herbert Christen

**Affiliations:** 1 Empa, Swiss Federal Laboratories for Materials Science and Technology, Laboratory for Applied Wood Materials, St. Gallen, Switzerland; 2 Empa, Swiss Federal Laboratories for Materials Science and Technology, Laboratory for Biointerfaces, St. Gallen, Switzerland; 3 Institute for Building Materials, ETH Zurich, Zurich, Switzerland; 4 Pavatex SA, Cham, Switzerland; Wageningen University, NETHERLANDS

## Abstract

Low-density wood fiber insulation boards are traditionally manufactured in a wet process using a closed water circuit (process water). The water of these industrial processes contains natural phenolic extractives, aside from small amounts of admixtures (e.g., binders and paraffin). The suitability of two fungal laccases and one bacterial laccase was determined by biochemical characterization considering stability and substrate spectra. In a series of laboratory scale experiments, the selected commercial laccase from *Myceliophtora thermophila* was used to catalyze the surface modification of thermo-mechanical pulp (TMP) using process water. The laccase catalyzed the covalent binding of the phenolic compounds of the process water onto the wood fiber surface and led to change of the surface chemistry directly via crosslinking of lignin moieties. Although a complete substitution of the binder was not accomplished by laccase, the combined use of laccase and latex significantly improved the mechanical strength properties of wood fiber boards. The enzymatically-treated TMP showed better interactions with the synthetic binder, as shown by FTIR-analysis. Moreover, the enzyme is extensively stable in the process water and the approach requires no fresh water as well as no cost-intensive mediator. By applying a second-order polynomial model in combination with the genetic algorithm (GA), the required amount of laccase and synthetic latex could be optimized enabling the reduction of the binder by 40%.

## Introduction

Wood fibers are one of the most important basic materials for the production of composite wood products. Fiberboards include hardboards, medium density fiberboards (MDF) and low-density boards. The latter are used as insulation, cladding, and roofing material for buildings as well as for sound absorption and similar applications. Any fiberboard can be classified according to particle size, production method, and density. The characteristic density for low-density boards is in the range of 100–400 kg m^−3^ [1]. During wet process industrial production, the fibers, slurried in water, are first temporarily stored in tubs and then formed to pressed fiber mats using a forming machine. After squeezing out water mechanically, the pressed fibers are transported to a drying tunnel and, finally, the required shape is given. A sustainable approach in fiberboard production consists of reducing the overall freshwater consumption by using a closed water circuit (industrial process water). Aside from admixtures (e.g., binders and paraffin), the process water contains a large amount of extracted natural phenolic compounds [2]. Synthetic petrochemical-based binders (e.g., latex) are added for quality improvement of individual products (mechanical strength properties) when the desired mechanical properties cannot be attained with the fiber material alone. Hence, the production costs of wood-based panels rose noticeably in recent years due to the continual increase in crude oil prices. Aside from wood, binders are the most expensive material components in wood fiber insulation boards. Striving for more environmentally friendly industrial production processes involves issues concerning the effects of harmful chemicals on health, and increasing cost and limited accessibility of fossil-derived chemicals and, products. Thus, a future-oriented fiberboard production industry should consider using sustainable raw materials, particularly concerning the type and the amount of binders.

The use of enzymatic systems for surface activation of wood particles to form binder-less or binder-reduced composites, particularly MDF, has been the subject of study for over 20 years [3–6]. Wood fiber-lignin is a three-dimensional amorphous polymer consisting of methoxylated phenylpropane structures, which can be modified by oxidases such as lignin peroxidases, manganese peroxidases, and laccases [7]. Among the mentioned enzymes, laccases are preferred because of their substrate specificity and their few requirements. In contrast to peroxidases, laccases do not require hydrogen peroxide as a co-substrate. Laccases are multi-copper containing oxidases (EC 1.10.3.2), widely distributed in fungi, higher plants, and bacteria and they oxidize numerous aromatic and non-aromatic compounds with a concomitant reduction of molecular oxygen to H_2_O [7]. Because of the laccase redox potential at the active site for substrate oxidation of approximately ≤ 0.8 V, their action is restricted, and substrates with higher redox potentials cannot be oxidized by laccases directly [8]. However, the substrate range can be mediated to secondary substrates via mediators. Mediators are substrates for laccases and after oxidation, they can oxidize a secondary substrate in solution away from the active site of the enzyme. The presence of such small molecular weight compounds expands the substrate range of laccase towards more recalcitrant compounds such as non-phenolic lignin units [9].

The objective of the present work was to elucidate the potential of the laccase-catalyzed surface modification of thermo-mechanical pulp (TMP) using industrial process water for the production of low-density wood fiber boards. Within the study process water and modified TMP were analyzed by Fourier transform infrared spectroscopy (FTIR-atr). The mechanical properties of binderless and binder-reduced wood fiber boards made from treated TMP were compared with conventional bonded wood fiber boards. Furthermore, a second-order polynomial model was employed in combination with the genetic algorithm (GA) to find the optimal laccase-substrate concentration for the production of low-density wood fiber boards. The results obtained are discussed in terms of effectiveness and applicability.

## Material and Methods

### Wood fibers and industrial process water

The wood fibers, the industrial process water, and the additives paraffin as well as the binder latex (styrene-butadiene) were provided by the Swiss company Pavatex SA. Softwood (containing approximately 70% w/w *Picea abies* and 30% w/w *Abies alba*) was defibrated into wood fibers by applying a TMP process. The process water samples were taken from the industrial plant in Fribourg, Switzerland.

### Laccases and mediators

The fungal laccases *Trametes versicolor* (f-Tve) and *Myceliophthora thermophila* (f-Mth) were purchased from Sigma-Aldrich and Novozymes, respectively. A novel CotA-type laccase from *Bacillus pumilus* (b-Bpu) was expressed and purified as described by Reiss *et al*. [10]. All enzyme substrates used for enzyme assays (2,2'-azino-bis(3-ethylbenzothiazoline-6-sulphonic acid [ABTS], acetosyringone [ACS], 4-hydroxybenzoic acid [HBA]) and other chemicals were supplied by Sigma-Aldrich.

#### Laccase assay

Laccase activity was measured as initial velocity of the oxidation of ABTS (2,2'-azino-bis(3-ethylbenzothiazoline-6-sulphonic acid), 3 mM) to its cation radical at room temperature (22–25°C) at pH 4.5 (citrate buffer 100 mM). The reaction mixture contained 700 μl buffer, 100 μl ABTS and 200 μl of the sample. Change in absorbance (*Δ*A) at 420 nm was recorded by UV-visible spectroscopy. Samples with very high activities were diluted 10-fold with 100 mM citrate buffer before using them in the assay. One volumetric activity unit (U) was defined as the amount of enzyme transforming 1 μmol of ABTS per min and the volumetric activities were calculated using an extinction coefficient (Ɛ) of 36000 mol^-1^ L cm^-1^, according to the following equation (*Δ*t in min, V in μL):

$$\text{Laccase activity }\left(U\;L^{-1}\right)=\frac{\Delta A}{\Delta t*\varepsilon }*\frac{V_{total,\;assay}}{V_{sample}}*dilution\;factor_{sample}$$ (1)

### Stability and substrate range of the laccases

To determine the suitability of the laccases for surface modification of TMP using process water, the laccases were biochemically characterized considering stability and substrate spectra.

Thermal stability was determined by incubating the different laccases in deionized water at temperatures ranging from 30°C to 80°C for various periods. After cooling to room temperature, the residual activity was measured using the ABTS standard assay. The thermal denaturation kinetics of the laccases was described by the exponential equation of a first-order reaction process [11]:

$$\frac{A_{r}}{A_{0}}=\text{exp}\left(-k_{d}*t\right)$$ (2)

where *A*_*r*_ is the residual laccase activity after incubation at a particular temperature, *A*_*0*_ is the initial laccase activity (room temperature), *k*_*d*_ is the rate constant (per time), and *t* is the duration of incubation. The laccase half-life (*t*_1/2_) at different temperatures was determined using the following equation:

$$t_{1/2}=\frac{0.693}{k_{d}}$$ (3)

The ability of the laccases to oxidize 20 different substrates (S1 Table) was investigated as described by Reiss *et al*. [12] with slight modifications. Laccase with a total activity of 0.12 U mL^−1^ was added to start the reaction solution containing 1 mM substrate. The reactions were monitored at different pH values (2.5, 4.5, 6.5 and 7.5) at average room temperature (22–25°C) by UV-visible spectroscopy in the wavelength scan mode from 200 to 900 nm after 0, 5, and 10 min. The enzyme substrate combinations that resulted in a clearly distinct altered absorbance were marked as (+), whereas unchanged spectra were marked as (−). In a small number of cases the classification was not clear, and these were marked as (+/-). Control reactions without laccase were performed in parallel to rule out effects from possible non-enzymatic oxidation.

The temperature and time dependency of laccases stability in the industrial process water was additionally determined. Each laccase (1 U mL^−1^) was added to 2 mL of process water and incubated for different periods at room temperature (22–25°C) and at 40°C. After the incubation, the residual percent activity was measured using the ABTS standard assay. In addition, the effects of possible inhibitors on laccase activity at different concentrations were determined by the enzyme assay using ABTS as a substrate. The laccase was pre-incubated with the additives used in the wet process (latex, paraffin) at different concentrations for 5 min at room temperature, and the percent activity was determined.

### Preparation and evaluation of wood fiber insulation boards

Wood fiber boards were produced in the laboratory at Empa, St. Gallen (batch 1: 10 by 10 by 2 cm [length by width by height]), and at the pilot plant of Pavatex, Fribourg (batch 2: 20 by 20 by 2 cm). Wood fibers were treated in a 2–2.5% (v/v) suspension of industrial process water. No pH adjustment was made. Laccase was added (20 U g^−1^ fiber dry substance) and the suspension was stirred for 1 min. To test the suitability of the mediators, 10 mM of ACS and HBA were additionally used (laccase-mediator system). After 1 min of laccase-fiber incubation, the binder latex (5% w/v) and optional paraffin (0.5% v/v) were added, and the reaction mixture was stirred. The suspension was incubated and stirred for a further 30 min at room temperature. After the incubation period, the process water was drained mechanically (i.e., vacuum pump, cold pressing) from the fibers. The produced fiber mat was dried for 24 h at 103°C in a dry oven. All experiments were performed in triplicate and repeated at least once. Wood fiber insulation boards were prepared from the following different treatments:

*Lat*: Wood fibers incubated in process water with latex

*Lac*: Wood fibers incubated in process water with laccase

*Lac-Lat*: Wood fibers incubated in process water with laccase and latex

*LMS*: Wood fibers incubated in process water with the laccase-mediator system (LMS1 = ACS, LMS2 = HBA)

In addition, further control experiments were performed in fresh tap water (~pH 7, 30–35°C).

The board samples were conditioned and equilibrated at 65% RH and 22 C° prior to testing. The European Standard EN 13171 served for specifications on the produced wood fiber insulation boards. The density of the boards was determined and for each treatment, the boards were tested for compression strength at 10% deformation and internal bond strength according to the European Standard EN 826 and 1607, respectively. Significant difference was evaluated by using Fisher’s-LSD (least significant difference) test with a significance level of *P* < 0.05.

### FTIR-atr analysis

The industrial process water of the wet process was evaporated, yielding a thick and concentrated solution, which was subsequently analyzed by FTIR-atr.

Wood fibers were treated as described above, and half of the treated fibers were exposed to leaching for 4 h, including two steps of water changing. After drying, the wood fibers were analyzed using a spectrophotometer FTS-6000 with golden gate single reflection diamond ATR P/N 183 10500 Series (Portsman Instruments AG, Biel-Benken, Switzerland). The size of the measuring area was 0.36 mm^2^ and the resolution of the equipment was set to 4 cm^−1^ with 32 average scans. The contact pressure of the fibers was set to 100 cN. The peak maxima were determined using the software Resolutions Pro 188 (Digilab, Holliston, 189 USA). Baseline corrections with asymmetric least squares smoothing and the Savitzky-Golay algorithm were performed for all spectra using Matlab Software (ver. 7.10.0 MathWorks, R2010a). Subsequently, the spectra were vector normalized according to following equation:

$$x_{i}=\frac{y_{i}}{\sqrt{\sum_{j=1}^{n}{\left(y_{i}\right)}^{2}}}$$ (4)

*y*_*i*_ = absorption value.

### Bioprocess optimization

To explore the effect of laccase and binder concentration on the response (internal bond strength) in the region of interest, a 5-level 2-factor central composite design (CCD) was performed aiming at the reduction of the enzyme and synthetic binder concentration.

A second-order polynomial model was defined to fit the response [13]. To assess the fitting and predictive accuracy of the model, the datasets were mathematically evaluated by calculating the following evaluation criteria: coefficient of determination (*R* ^*2*^), root mean squared error (RMSE), mean relative percentage error (MRPE), mean absolute percentage error (MAPE) [14], and the proportion of the relative error (pRE); [15].

Genetic algorithm optimization was performed using the ‘ga’ function of Matlab and with the polynomial model as the fitness function. The upper and lower bounds of the input variables (genes) were determined (*x*_*i*_^*L*^ < *x*_*i*_ < *x*_*i*_^*U*^). *x* denotes the experimental conditions, and *x*_*i*_^*L*^ and *x*_*i*_^*U*^ represent the lower and upper bound on *x*_*i*_ (Table 1). The input parameters of the ‘ga’ function were as described in Schubert *et al*. [16].

**Table 1:** Experimental range and level of central composite design for independent input variables in coded terms.

Factor	Variable	Unit	Range and level of actual and coded values				
			−1.4142	−1	0	+1	+1.4142
*X*_*1*_	Laccase	U g^−1^	0.24	0.65	1.65	2.65	3.1
*X*_*2*_	Latex	%	0.17	1	3	5	5.8

## Results and Discussion

### Stability and substrate range of the laccases

The thermal deactivation constant *k*_*d*_ h^−1^ and the half-life *t*_1/2_ of the laccases are shown in Table 2. It was found that the thermal stability of the laccases was approximately b-Bpu > f-Mth > f-Tve. In particular, the Bpu laccase showed the lowest thermal deactivation constant and the highest half-life at all temperatures (Table 2). Furthermore, after 1 h at 80°C a residual laccase activity of > 60% (*k*_*d*_ h^−1^ 0.502) could be detected for Bpu, whereas for the fungal laccases Mth (*k*_*d*_ h^−1^ 3.107) and Tve (*k*_*d*_ h^−1^ 7.857) no residual activity could be observed after 1 h at 80°C. Reiss *et al*. [10] showed similar results regarding the high-temperature tolerance of the *B*. *pumilus* laccase (Bpu). Comparing the two fungal laccases, Mth laccase was more temperature stable than the Tve laccase, as indicated by the lower thermal deactivation constant *k*_*d*_ h^−1^ (Table 2).

**Table 2:** Thermal deactivation constant (*k*_*d*_ h^−1^) and half-life (*t*_*1/2*_) of the laccases.

	f-Tve			f-Mth			b-Bpu		
°C	*k*_*d*_ (h^−1^)	*R* ^*2*^	*t*_1/2_ (h)	*k*_*d*_ (h^−1^)	*R* ^*2*^	*t*_1/2_ (h)	*k*_*d*_ (h^−1^)	*R* ^*2*^	*t*_1/2_ (h)
**40**	0.049	0.974	14.3	0.006	0.998	113.5	0.003	0.999	280.5
**50**	0.068	0.982	10.2	0.055	0.999	12.6	0.019	0.997	35.9
**60**	0.152	0.938	4.57	0.115	0.999	6.31	0.036	0.998	19.2
**70**	3.079	0.957	0.23	2.366	0.931	0.31	0.138	0.782	5.04
**80**	7.857	0.844	0.08	3.107	0.959	0.22	0.502	0.922	1.38

Tve = *Trametes versicolor*

Mth = *Myceliophthora thermophila*

Bpu = *Bacillus pumilus*

f = fungal Laccase

b = bacterial Laccase

**Fig 1:**
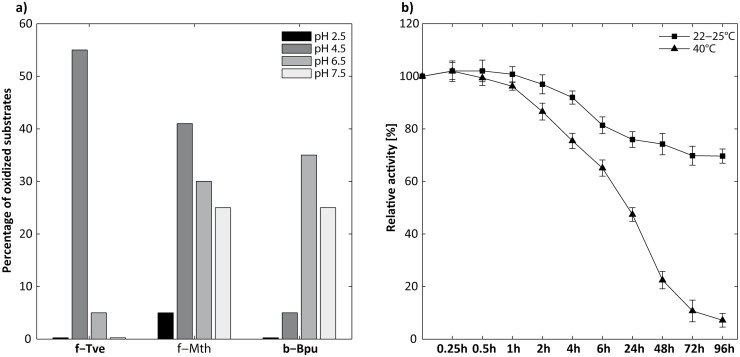
Oxidation potential and laccase stability. **a)** Activity of the laccases (%) towards synthetic and natural compounds (S1 Table) at different pH. f-Tve: *Trametes versicolor*; f-Mth: *Myceliophthora thermophila*; b-Bpu: *Bacillus pumilus*. **b)** Long-time effects of the industrial process water on the f-Mth laccase activity at room temperature (∎) and 40°C (▲). f-Mth: *Myceliophthora thermophila*.

The enzymatic oxidation potential was tested by using 20 synthetic and natural compounds as potential substrates at different pH values (2.5–7.5), comprising aromatic carboxylic acids, aromatic alcohols, aromatic aldehydes, aromatic amines, and aromatic azo compounds. In Fig 1a, the activity (in percent) of the different laccases towards the different compounds is shown. *Trametes versicolor* (f-Tve) was found to have the broadest substrate spectrum considering the selected compounds at pH 4.5, which might be due to the high redox potential of this enzyme (E^0^ = 0.79 V) [17]. However, the capability to oxidize the substrates is strongly dependent on the pH, as shown by the significant reduction in the activity at lower or higher pH (2.5, 6.5, and 7.5). The *M*. *thermophila* laccase turned over most of the substrates at pH 4.5, but the activity was slightly reduced compared to the f-Tve laccase, probably due to the lower redox potential (E^0^ = of 0.5 V) [18]. Nonetheless, f-Mth showed at all pH values tested a considerably high oxidation activity towards the selected substrates, particularly at basic pH (Fig 1a). The bacterial laccase (b-Bpu) revealed the smallest substrate range in this study when compared with the fungal laccases and the favorable pH was found to be around the neutral pH region (6.5 and 7.5), whereas the activity was negligible at acidic pH 4.5 and particularly at 2.5.

The technical requirements for an efficient use of the laccases are defined by the characteristics of the process water. The FTIR results showed that the industrial process water contains high amounts of polysaccharides and natural phenolic extractives, along with small amounts of admixtures (e.g., binder; data not shown). This is in good agreement with the work of Örså *et al*. (1997) who described that the main dissolved components in mechanical pulping are carbohydrates (hemicellulose) and lignins (phenolic compounds). Moreover, the average solid fraction of the process water used, determined after evaporation, was found to be 1.4% (w/v). The pH and temperature of the industrial process water can vary between 4–6 and 30–60°C respectively, with an average pH of approximately 4.5 and an average temperature of 45°C.

Based on these characteristics, thermal stability and particularly high activity towards different compounds under varying pH (4–6) are the main requirements for laccase-initiated reactions using industrial process water.

Although the bacterial laccase (b-Bpu) showed the highest thermal stability in this study, the enzyme was not suitable for laccase-initiated reactions using process water because of the limited substrate range (lowest oxidative capabilities) and low activity, particularly in the acidic pH range (4–5) of the process water. The *M*. *thermophila* laccase (f-Mth), when compared to the *Trametes* laccase (f-Tve), revealed higher activity at elevated temperatures and showed a broader substrate range at varying pH values. Moreover, as illustrated in Fig 1b, the Mth laccase was active and stable in the process water: a residual activity of 69% (*k*_*d*_ h^−1^ 0.0038) could be detected even after an incubation time of 96 h at room temperature. The Mth laccase stability at 40°C in process water (*k*_*d*_ h^−1^ 0.0274) was reduced compared to its thermal stability in deionized water at room temperature or at 40°C (*k*_*d*_ h^−1^ 0.006). The higher deactivation constant at 40°C in process water (solid content of 1.37% w/v) might be due to shearing and aggregation, which may negatively affect the stability of the enzyme. Additionally, the high concentration of other compounds in the process water may be involved in laccase denaturation like admixtures. However, the fiber incubation time in the process water is 30 min, a period in which the enzyme is highly stable; moreover, chemical substances present in the process water showed no inhibitory effect. Overall, the enzyme is compatible with the process water and reusable for the modification of the TMP in a closed water circuit. Future studies involving immobilization techniques and enzyme engineering, particularly directed evolution of structure-guided site-directed mutagenesis, might be promising to obtain laccase variants exhibiting increased oxidation potential and stability in process water [19].

### Preparation and evaluation of wood fiber insulation boards

Densities in the range of 100–400 kg m^−3^ are characteristic for low-density fiberboards like insulation products. In our case, the average fiberboard density was found to be 123.63 kg m^-3^, and according to Fisher’s-LSD test, no significant density differences (*P ≥* 0.05) between the different fibers could be observed (*Lac*: 125 ± 4.20 kg m^−3^; *Lat*: 120.75 ± 10.25 kg m^−3^; *Lac-Lat*: 125.14 ± 10.24 kg m^−3^).

**Fig 2:**
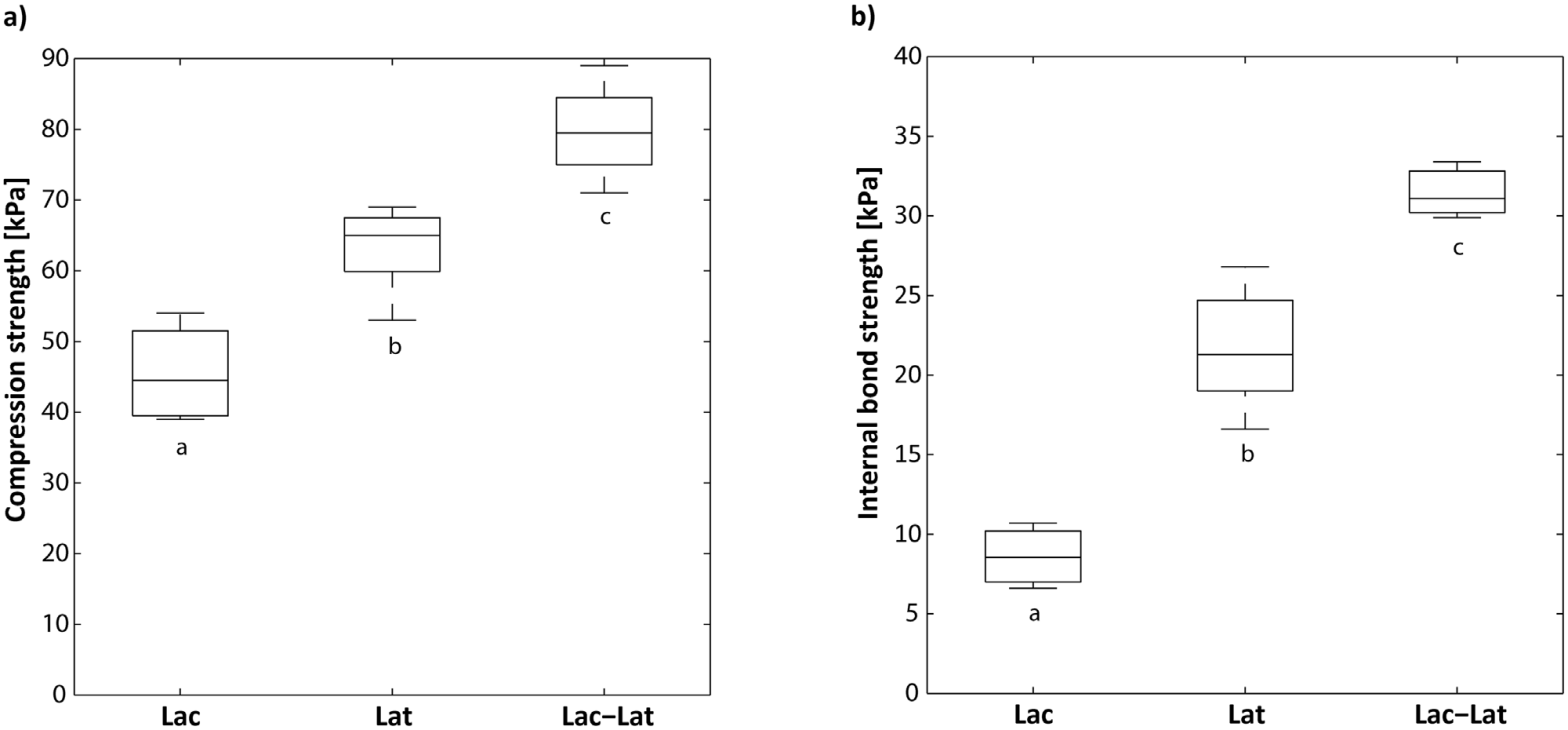
Influence of laccase on the mechanical properties of the wood fiber boards (~125 kg m^-3^). **a)** Compression strength at 10% deformation and **b)** Internal bond strength. *Lac*: 20 U g^−1^ fiber; *Lat*: 5% latex; *Lac-Lat*: 20 U g^−1^ fiber + 5% latex. Letters denote significant differences between the wood-fiber treatments after Fisher’s LSD test (*P* ≤ 0.05).

The key mechanical properties of wood fiber insulation boards are compression strength and, particularly, internal bond strength. Therefore, the evaluation of the produced insulation boards focused on these mechanical parameters. The laccase-only (20 U g^−1^ fibers) treated boards (*Lat*) revealed significant lower mechanical strength properties compared to the reference boards (*Lat*) with 5% (w/v) latex (Fig 2). This is remarkable, as a number of papers have been published on the successful manufacture of binderless fiberboards (MDF and HDF) made by laccase oxidized fibers [3, 4, 6, 20, 21]. The discrepancy may be founded in the higher density of MDF and HDF (> 600 kg m^−3^) compared to the low-density boards produced in this work (~ 125 kg m^−3^) and in the use of high temperature 180–200°C (hot press) for the MDF and HDF production. Pressing at high forces and temperature for long periods can create water-resistant bonds between wood fibers that improve the mechanical performances [22, 23]. Moreover, the above-mentioned authors used a defined buffer system with a favorable pH for the laccase reactions. For the application described here, this approach would result in increased fresh water consumption and was, therefore, not considered.

Although laccase only (*Lat*) was not suitable for the production of low-density insulation boards, the boards made from laccase-latex treated fibers in process water (*Lac-Lat*) revealed significantly higher compression strength and internal bond strength (*P* < 0.05) compared to the reference (5% w/v-latex boards, *Lat*), as shown in Fig 2.

**Fig 3:**
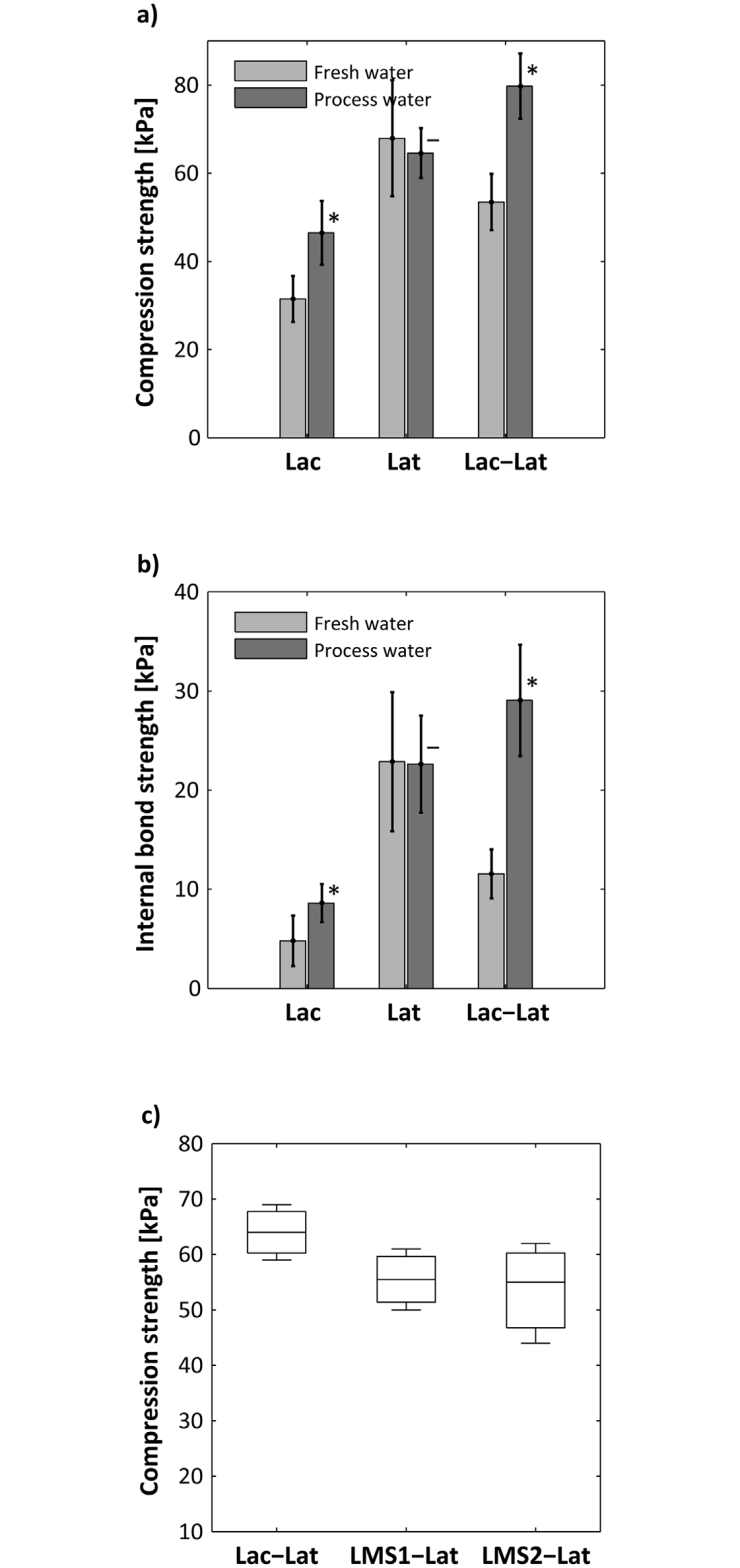
Influence of the process water on the mechanical properties of the wood-fiber boards (~125 kg m^-3^). **a)** Compression strength at 10% deformation, **b)** Internal bond strength. *Lac*: 20 U g^−1^ fiber; *Lat*: 5% latex; *Lac-Lat*: 20 U g^−1^ fiber + 5% latex, and **c)** Effect of the laccase mediator system (LMS) on the compression strength at 10% deformation. *LMS1-Lat*: 20 U g^−1^ fiber + 10 mM ACS (acetosyringone) + 5% latex; *LMS2-Lat*: 20 U g^−1^ fiber + 10 mM HBA (4-hydroxybenzoic acid) + 5% latex. *Significant difference between process water and fresh water concerning mechanical strength properties (t-test, *P* ≤ 0.05).

It turned out that for the treatment of wood fibers in process water, laccase-catalysis was crucial for obtaining improved mechanical strength properties. The effectiveness of *Lac* and *Lac-Lat* is significantly reduced in batches containing fresh water when compared to one containing process water, whereas for *Lat* no significant difference was detectable (Fig 3a and 3b). As already mentioned, the process water contains a large amount of natural phenolic extractives [2]. Kudanga *et al*. [24] showed in his review that polyphenols could successfully be grafted onto lignocellulose by using laccases to improve certain material properties. Laccases catalyze the one-electron oxidation of substrates at the T1 Cu-site, coupled to the reduction of oxygen to water at the trinuclear Cu-site [7]. Zhou *et al*. [25] could show in their study of laccase-catalyzed oxidation of wood fibers, that reactive oxygen species (ROS) e.g. O_2_^•–^ and ^•^OH are the main free radical intermediates and generated, aside from phenoxy radicals, during enzyme-lignin interaction. The reactive substrate radicals upon laccase mediated oxidation can then undergo a number of non-enzymatic reactions including covalent coupling to dimers, oligomers, and polymers through C-C, C-O, and C-N bond formation [24]. In the case of laccase catalysis in process water, the enzyme catalyzes the oxidation of the wood fiber surface, leading to subsequent surface modification through intermolecular reactions thereof. Moreover, the diverse phenolic extractives within the process water might be oxidized to radicals and bound to the wood fiber surface via crosslinking events. Through such events the chemical composition and the surface energy of the wood fiber surface was changed [26–28]. For instance, Felby *et al*. [26] demonstrated by wetting analysis that fibers treated with lignin and laccase showed a markedly increased hydrophobicity.

**Fig 4:**
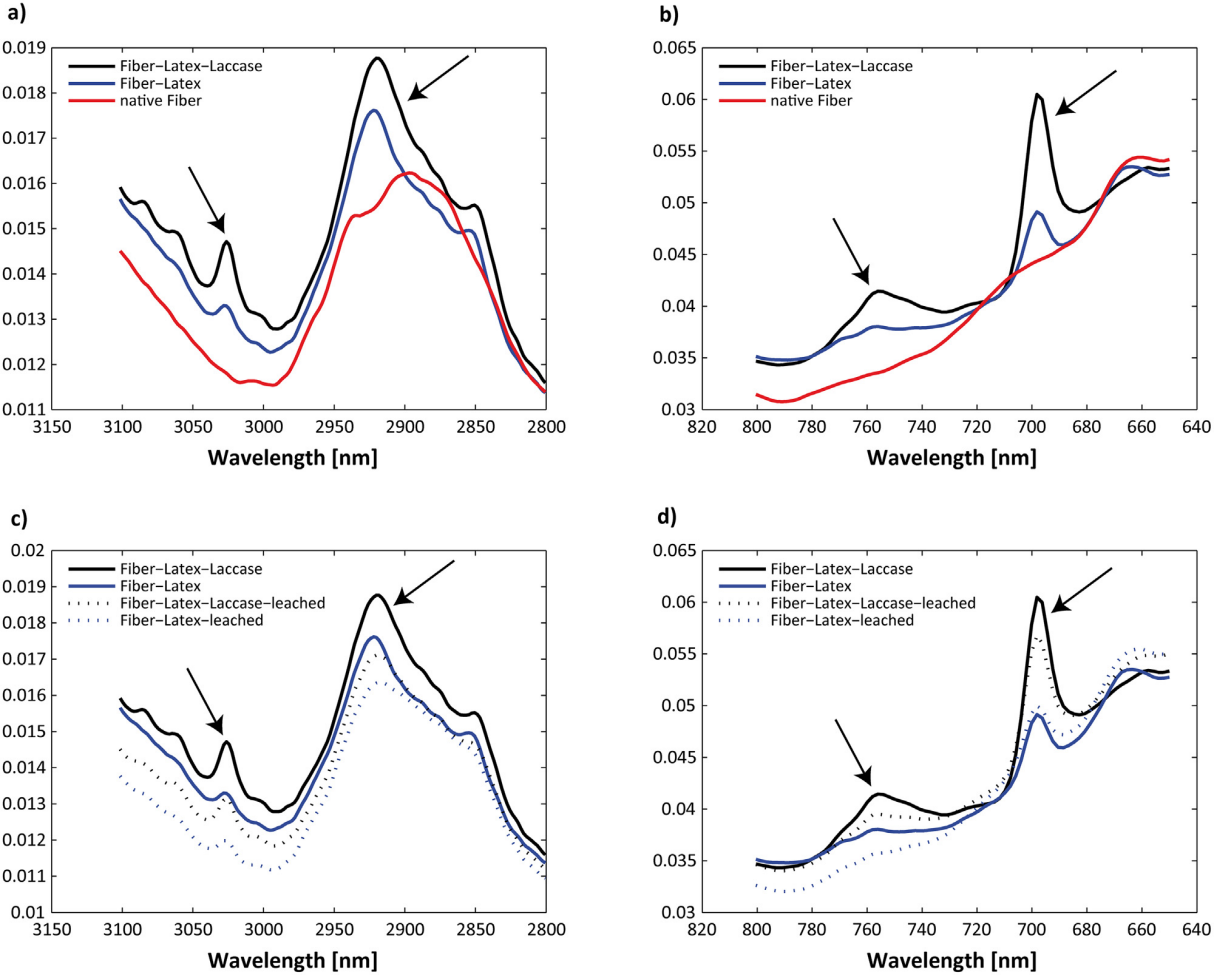
FTIR-atr spectra of treated wood fibers. **a–b)** Characteristic latex peaks were detectable compared to native wood fibers (arrows), **c–d)** Even after leaching procedure the laccase treated fibers showed characteristic peaks deriving from binder (arrows).

The enhanced wood fiber surface energy most likely led eventually to an improvement of the fiber-binder adhesion, which could be observed from the improved mechanical strength properties and the FTIR-atr analysis (Fig 4). FTIR spectra of native wood fibers and latex-treated fibers revealed some typical absorption peaks for latex at 698, 756, 2922 and 3028 cm^−1^ (Fig 4a and 4b). These characteristic latex peaks were considerably more pronounced in the laccase-treated fibers than in the reference fibers (latex only), indicating that more latex could adhere to the fiber surface after laccase treatment. Moreover, even after a 4 h leaching procedure of the wood fibers, the laccase-treated fibers revealed remarkable better fiber-binder interaction compared to the reference (Fig 4c and 4d). Although the detailed fiber-binder interaction is unknown, it cannot be ruled out, that the laccase-catalyzed generation of radicals on the fiber surface may lead to covalent bonds via radial coupling of lignin moieties with the polymer latex (styrene- butadiene). Further studies have to elucidate the detailed fiber-binder mechanisms.

Because of their redox potential (0.5–0.8 V at the active site of the enzyme), the laccases can readily only oxidize phenolic building blocks of lignin units or derivatives thereof [29]. As already described, the substrate range can be extended to secondary substrates through laccase mediator systems. Aside from synthetic mediators, naturally-derived compounds, e.g. acetosyringone (ACS) and 4-hydroxybenzoic acid (HBA), have been used as mediators for different applications [8]. In particular, HBA has been used successfully by Euring *et al*. [20] for the manufacturing of medium-density wood fiber boards. Interestingly, in the present work, the additional use of ACS and HBA within a laccase-mediator system led to no additional effect on the process performance or mechanical properties of the wood-fiber boards. Neither the compression strength nor the internal bond strength could be improved by the LMS, as illustrated exemplarily for compression strength in Fig 3c. One possible explanation could be that some phenolic extractives in the process water might function as mediators and improve the reactivity, making the application of additional compounds unnecessary [29, 30]. As discussed earlier, the main dissolved components in mechanical pulping of Norway spruce wood are carbohydrates, particularly phenolic substances such as coniferyl alcohol, vanillin, and vanillic acid [2, 31]. Camarero *et al*. [32] and Hassingboe *et al*. [28] demonstrated that lignin-derived compounds, amongst others, vanillin and vanillic acid, and water soluble components of Norway spruce thermo-mechanical pulp fibers can function as small natural molecules, which improve laccase-based processes. Further studies are needed to reveal detailed mechanisms of laccases (and potential mediators) in process water. Most importantly, the study showed that no cost-intensive commercial mediator has to be applied in the enzymatic system using industrial process water for fiberboard production.

### Bioprocess optimization

If the enzyme laccase was to be applied as a technical compound in bulk quantities in the forest product industry, the required amount of the enzyme (as cost factor), has to be minimized. Moreover, since the application of laccase in process water resulted in significantly improved strength properties of the 5% (w/v)-latex bonded boards, it should be possible to reduce the amount of the petrochemical-based binder latex. Therefore, a polynomial model in combination with the genetic algorithm (GA) was employed to find the optimal laccase-substrate concentration. The genetic algorithm is a combinatorial optimization technique to search for an optimal value of a complex objective function by simulation of the biological evolutionary process based on crossover and mutation. GAs have been used successfully in a wide variety of applications [33].

The following second-order regression equation was obtained to explain wood fiber board internal bond strength in terms of the initial values of the experimental conditions:

$$Y=\;3316\;+\;53.1X_{1}+\;180.2X_{2}-\;409.8X_{1}{}^{2}-\;486.6X_{2}{}^{2}-\;130X_{1}X_{2}$$ (5)

where *Y* is the internal bond strength as response and *X*_*1*_ and *X*_*2*_ are concentrations of laccase and latex, respectively.

**Fig 5:**
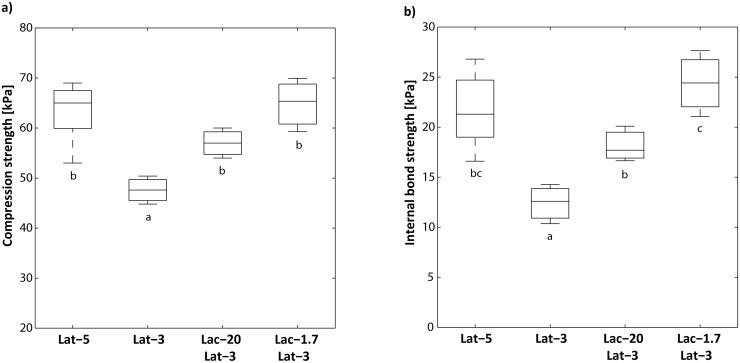
Influence of the optimized laccase concentration on the mechanical properties of the wood fiber boards (~125 kg m^-3^). Bioprocess optimization was carried out by a second-order polynomial model in combination with the genetic algorithm (GA). **a)** Compression strength at 10% deformation and **b)** Internal bond strength. *Lat-5*: 5% latex; *Lat-3*: 3% latex; *Lac-20 Lat-3*: 20 U g^−1^ fiber + 3% latex; *Lac-1*.*7 Lat-3*: 1.7 U g^−1^ fiber + 3% latex. Letters denote significant differences between the wood-fiber treatments after Fisher’s LSD test (*P* ≤ 0.05).

The analysis revealed an *F*-value of 13.68 and a *P*-value of 0.0024, indicating that the second-order polynomial model was significant. To verify the model’s adequacy, several indices also were estimated. In the present work, the calculated coefficient of determination (*R* ^*2*^) was 0.984. Furthermore, the root mean squared error (*RMSE* = 0.425), mean relative percentage error (*MRPE* = −0.099), mean absolute percentage error (*MAPE* = 2.612), and the proportion of the relative error (*pRE* = 1) revealed that the second-order polynomial model was considerably robust and reliable, thus appropriate to be used as a fitness function for GA. By applying the GA methodology, the synthetic binder amount could be reduced by 40% for the production of wood fiber insulation boards with constant or slightly better mechanical properties compared to the 5% (w/v)-latex boards (Fig 5). In addition, the laccase concentration was optimally down-regulated to 1.7 U g^−1^ fibers (0.04 U mL^−1^). This indicates a truly catalytic process and together with the stability of the applied laccase in process water, it is an important criterion for both the efficiency and the sustainability of the developed process. According to Hollmann and Arends [34], one way to influence the outcome of radical polymerization (crosslinking) reactions is the change of the biocatalyst concentration. At very high enzyme concentration, the initiation rate can be so dominant that only activated oligomers are formed that do not further polymerize, while enzyme concentrations that are too low result in low conversions e.g. due to enzyme inactivation [34]. These results support the finding that the optimal laccase concentration is an important aspect in achieving optimal effectiveness of laccase-catalyzed surface modification of TMP in process water. However, because the industrial process water characteristics can vary considerably over time, the results concerning the laccase concentration should be considered as approximate values. Nevertheless, the approach developed here is certainly important for simulation of economic wood fiber insulation board production with respect to reducing the cost factors in scale-up studies.

## Conclusion

Improvements in wood fiber adhesion are desirable in terms of performance enhancement, while using only a limited amount of adhesives. To achieve these goals biocatalysts enable access to green and sustainable production processes. In the present study, the effect of laccase-catalyzed fiber surface modification using industrial process water on the performance of wood fiberboards was investigated.

The present work shows that although the synthetic binder latex could not be completely substituted through laccase, the integration of the enzyme in a wet process using industrial process water had the following advantages: (1) the enzyme catalyzes the covalent binding of the phenolic compounds of the process water onto the wood fiber surface, thus, increasing the fiber-surface energy, (2) the combined application of the enzyme with the standard amount of latex resulted in significant improvement of the mechanical properties, (3) it is possible to reduce considerably the required amount of the fossil-based binder, (4) the enzyme is stable in the process water, (5) no fresh water, and no cost-intensive mediator is needed. Moreover, laccase catalysis opens possibilities to substitute synthetic binders with naturally derived biopolymers.

## Supporting Information

S1 Table Selected synthetic and natural compounds for the laccase substrate screen. (DOCX)
