# Supplementary material for: Laccase-Catalyzed Surface Modification of Thermo-Mechanical Pulp (TMP) for the Production of Wood Fiber Insulation Boards Using Industrial Process Water
Source: PLoS One. 2015 Jun 5;10(6):e0128623. doi: 10.1371/journal.pone.0128623 (PMC4457874; doi:10.1371/journal.pone.0128623)
Supplement: S1 Table — (DOCX) [file pone.0128623.s001.docx]

**Supplementary Table**

**S1 Table:** Selected synthetic and natural compounds for the laccase substrate screen.

| **Substance** |  | **p-Tve** | **p-Mth** | **b-Bpu** | **Substance** |  | **p-Tve** | **p-Mth** | **b-Bpu** |
| --- | --- | --- | --- | --- | --- | --- | --- | --- | --- |
| 2-Methoxyphenol | **pH 2.5** | - | - | - | trans-4-Hydroxy-cinnamic acid | **pH 2.5** | - | - | - |
|  | **pH 4.5** | + | + | - |  | **pH 4.5** | +/- | - | - |
|  | **pH 6.5** | +/- | + | +/- |  | **pH 6.5** | - | - | - |
|  | **pH 7.5** | - | + | + |  | **pH 7.5** | - | - | - |
| 2,6-Dimethoxyphenol | **pH 2.5** | - | - | - | 2,5-Dimethyl aniline | **pH 2.5** | - | - | - |
|  | **pH 4.5** | + | + | - |  | **pH 4.5** | +/- | +/- | - |
|  | **pH 6.5** | + | + | + |  | **pH 6.5** | +/- | - | - |
|  | **pH 7.5** | - | + | + |  | **pH 7.5** | - | - | - |
| 3,4-Dimethoxybenzylalcohol | **pH 2.5** | - | - | - | 4-Hydroxy-3,5-dimethoxy-cinnamic acid | **pH 2.5** | +/- | + | - |
|  | **pH 4.5** | - | - | - |  | **pH 4.5** | + | + | - |
|  | **pH 6.5** | - | - | - |  | **pH 6.5** | - | + | + |
|  | **pH 7.5** | - | - | - |  | **pH 7.5** | - | + | + |
| 1,2-Benzenediol | **pH 2.5** | - | - | - | trans-4-Hydroxy-3-methoxy-cinnamic acid | **pH 2.5** | - | - | - |
|  | **pH 4.5** | + | +/- | - |  | **pH 4.5** | + | + | - |
|  | **pH 6.5** | +/- | - | + |  | **pH 6.5** | - | + | + |
|  | **pH 7.5** | - | - | +/- |  | **pH 7.5** | - | + | + |
| 4-Hydroxy-3-methoxybenzaldehyde | **pH 2.5** | - | - | - | 2-(4-Hydroxy-phenyl)ethylamine | **pH 2.5** | - | - | - |
|  | **pH 4.5** | - | - | - |  | **pH 4.5** | - | +/- | - |
|  | **pH 6.5** | - | - | - |  | **pH 6.5** | - | - | - |
|  | **pH 7.5** | - | - | - |  | **pH 7.5** | - | - | - |
| 4-Hydroxy-phenoxyacetic acid | **pH 2.5** | - | - | - | 4-Hydroxy-3-methoxybenzoic acid | **pH 2.5** | - | - | - |
|  | **pH 4.5** | + | - | - |  | **pH 4.5** | - | - | - |
|  | **pH 6.5** | - | - | - |  | **pH 6.5** | - | - | - |
|  | **pH 7.5** | - | - | + |  | **pH 7.5** | - | - | - |
| 4-Hydroxy-3-methoxybenzylamine | **pH 2.5** | - | - | - | 3,4-Dihydroxy-phenylalanine | **pH 2.5** | - | - | - |
|  | **pH 4.5** | - | - | - |  | **pH 4.5** | + | - | - |
|  | **pH 6.5** | - | - | - |  | **pH 6.5** | +/- | +/- | + |
|  | **pH 7.5** | - | - | - |  | **pH 7.5** | - | +/- | +/- |
| 3,5-Dimethoxy-4-hydroxybenzoic acid | **pH 2.5** | - | - | - | N-Methyl-phenethylamine | **pH 2.5** | - | - | - |
|  | **pH 4.5** | + | + | - |  | **pH 4.5** | +/- | - | - |
|  | **pH 6.5** | - | + | +/- |  | **pH 6.5** | - | - | - |
|  | **pH 7.5** | - | + | +/- |  | **pH 7.5** | - | - | - |
| 4-Hydroxy-3,5-dimethoxybenzaldehyde | **pH 2.5** | - | - | - | 4-Hydroxybenzoic acid | **pH 2.5** | - | - | - |
|  | **pH 4.5** | + | + | - |  | **pH 4.5** | - | - | - |
|  | **pH 6.5** | - | + | +/- |  | **pH 6.5** | - | - | - |
|  | **pH 7.5** | - | +/- | +/- |  | **pH 7.5** | - | - | - |
| 3,4,5-Trihydroxy-benzoic acid | **pH 2.5** | - | - | - | 1,2,3-Trihydroxy-benzene | **pH 2.5** | - | +/- | +/- |
|  | **pH 4.5** | + | + | - |  | **pH 4.5** | + | + | + |
|  | **pH 6.5** | - | - | + |  | **pH 6.5** | +/- | +/- | + |
|  | **pH 7.5** | - | - | - |  | **pH 7.5** | - | - | - |

Activity towards a substrate based on a change of absorbance is given as (+), no activity as (-) and ambiguous activity (+/-) when not clear.

Tve = *Trametes versicolor*

Mth = *Myceliophthora thermophila*

Bpu *= Bacillus pumilus*

f = fungal Laccase

b = bacterial Laccase
